# Super Bolus—A Remedy for a High Glycemic Index Meal in Children with Type 1 Diabetes on Insulin Pump Therapy?—A Randomized, Double-Blind, Controlled Trial

**DOI:** 10.3390/nu16020263

**Published:** 2024-01-16

**Authors:** Emilia Kowalczyk-Korcz, Magdalena Dymińska, Agnieszka Szypowska

**Affiliations:** 1 Department of Pediatric Diabetology, The Children’s Clinical Hospital Named after J.P. Brudziński, University Clinical Center of the Warsaw Medical University, 02-091 Warsaw, Poland; magdalena.dyminska@uckwum.pl (M.D.); agnieszka.szypowska@wum.edu.pl (A.S.); 2 Department of Pediatrics, Medical University of Warsaw, 02-091 Warsaw, Poland

**Keywords:** super bolus, postprandial glycemia, high glycemic index meal, meal bolus

## Abstract

Background: This study aimed to compare whether a super bolus (SB) is a more efficient strategy than a normal bolus (NB) for high glycemic index (h-GI) meals in children with type 1 diabetes (T1D). Methods: A randomized, double-blind, crossover trial with an allocation ratio of 1:1, registered at ClinicalTrials.gov (NCT04019821). 72 children aged 10–18 years with T1D > 1 year, and on insulin pump therapy > 3 months were included. As an intervention, they ate a h-GI breakfast for the two following days and receive a prandial insulin bolus either in the form of SB or NB. Results: The SB group had lower glucose values during the observation time and lower glucose levels in 90th min (primary end point). The median time in range was also higher after SB. At the same time, more hypoglycemic episodes and a higher time below range were noted in this group. Almost 90% of them were the threshold value for initiating treatment for hypoglycemia and occurred near the end of observation period. More hyperglycemic episodes and over twice as much time in hyperglycemia were noted after NB. Conclusions: Super bolus is an effective strategy to avoid postprandial hyperglycemia but the basal insulin suspension should be longer to avoid hypoglycemia (f.ex. 3 h).

## 1. Introduction

Postprandial hyperglycemia (PPH) is highly prevalent among people with type 1 diabetes (T1D). High glycemic index (h-GI) meals are mainly responsible for PPH; thus, many clinical practice guidelines recommend a low glycemic index (l-GI) diet for people with T1D [1,2,3]. L-GI patterns are associated with improved glycemic control as well as reduced cardiometabolic risk [4]. 

A poor adherence to a healthy diet is an everyday struggle and a difficult part of diabetes management among children [3]. Low-carbohydrate or ketogenic diets have recently become very popular because they ease the glycemic spikes, and provide greater glycemic stability and lower HbA1c values [3]. Despite the positive effects, the International Society for Pediatric and Adolescent Diabetes (ISPAD) do not recommend these types of diets for children because they are potentially inadequately nourished and may lead to growth and pubertal problems [1,3]. The possible strategy in this group is not to exclude the h-GI carbohydrates from the diet but to properly balance the consumption of h-GI and l-GI meals [3]. 

What do we know about the direct consequences of the ingestion of h-GI food now? The glucose level after consumption increases rapidly, leading to hyperglycemia (typically within 60 min–2 h) followed by a sudden drop in the glucose level [1,5,6,7]. Products with h-GI compared to l-GI, which contain the same amount of carbohydrates, cause a 20% higher area under the blood glucose curve (AUC) [7,8]. That is why these products require separate strategies to reduce glycemic spikes caused by them [1]. 

The known strategy to reduce postprandial hyperglycemia is the consumption of fat and protein 15 min before a h-GI meal among children [9]. Another recommendation is a pre meal bolus, typically taken 15–20 min before meal, but it is insufficient in case of h-GI [1]. The type of insulin is also a factor that influences the postprandial glycemia. Previous studies indicate that neither glulisine nor aspart insulin provide a stable and in-range glycemic profile [10]. A new insulin formulation-faster insulin, aspart, has a potential effect on reducing PPH in the youngest groups and adults, though this action was not confirmed among adolescents [11,12,13]. Data from adults indicate that a strategy of an additional dose of insulin to h-GI meals was studied before. Increasing the meal bolus dose by 30% resulted in a decrease in postprandial glycemic values without increasing the rate of hypoglycemia, while the percentage of hyperglycemia remained high [14]. 

It remains a clinical challenge to adjust the insulin dose and bolus pattern for h-GI meals. The goal of insulin therapy is to mimic as much as possible the physiological secretion of insulin. That is why in recent years the idea of the super bolus (SB) strategy is popular in context of h-GI products. The assumption is that SB boosts the prandial insulin to avoid glycemic spikes whilst removing the basal insulin to prevent postprandial hypoglycemia [5]. Many diabetes organizations recommend this strategy to deal with h-GI products, but the definition is not clear (how much the meal bolus should be increased and how long basal insulin should be removed) [15,16]. There is a lack of clinical studies concerning this type of bolus; available reports refer only to the in-silico model [17,18].

We defined the super bolus by using the following criteria:

− 50% increase in prandial bolus dose,
− removal of the basal insulin for 2 h post meal.

The proposed solution is based on reports from diabetes organizations, clinical practice, and our patients’ best experience. The purpose of this study was to compare whether SB is a more efficient strategy than the normal bolus (NB) for h-GI meals in children with T1D using continuous subcutaneous insulin infusion (CSII). 

## 2. Materials and Methods

This study was designed as a randomized, double-blind crossover with an allocation ratio of 1:1 and was conducted between January 2020 and January 2023 in our diabetology department. The ethics committee of the Medical University of Warsaw approved the study KB/25/2019. The trial was registered at ClinicalTrials.gov (NCT04019821) before the first patient was included. The survey was conducted in accordance with the CONSORT statement for reporting randomized trials [19]. The study protocol with a detailed course of the study was published in BMC Trials [20]. 

The study included children aged 10–18 years with a T1D duration of longer than 1 year, on insulin pump therapy for more than 3 months. Inclusion criteria were constant and were not changed during the course of the study. We excluded patients with celiac disease, diabetes-related complications (e.g., nephropathy), insulin faster aspart users, and those with obesity (defined as body mass index, BMI ≥ 95th percentile for children and teenagers of the same age and sex). All participants were hospitalized before and during the study. The study began with a run-in period lasting about one week. During that time, patients had their daily glycemic profile evaluated based on which individualized insulin-to-carb ratios (ICR) were established. The insulin doses were adjusted to meet target fasting and postprandial glucose levels. Only when normoglycemia was achieved for 2 following days after breakfast, did we assume that ICR is properly adjusted and the participant was included to the study. The fasting blood glucose level to enter the study was ≤130 mg/dL (>7.2 mmol/L), according to ISPAD pre-meal targets guidelines [21]. Before the study began, participants have applied the continuous glucose monitoring (CGM) system—Enlite™ sensor with a MiniLink™ Transmitter and a MiniMed^®^ Paradigm VEO™ insulin pump (Medtronic MiniMed, Northridge, CA, USA). As an intervention, they ate a h-GI breakfast for the two following days and the order was randomized to receive a prandial insulin bolus either in the form of a super bolus (intervention, SB group) or a normal bolus (control, NB group). We used a blocked randomization (blocks of four). The randomization list was generated using the program (V.3.1.4, StatsDirect, Chesire, UK). All participants and investigators were blinded. The investigator received randomly-generated treatment allocations within sealed opaque envelopes. Once a participant entered the trial, an envelope was opened and the allocated treatment regimen was applied. A nurse not involved in the trial programmed the meal bolus in compliance with the patient’s allocation found in the sealed envelope and calculated dose of insulin. The screen of the insulin pump was covered by a piece of black tape. The h-GI breakfast consisted of breakfast cereal: chocolate cornflakes (50 g carbohydrates) with added 2% cold milk (10 g carbohydrates). SB was defined as the 50% increase in prandial insulin dose (150%) compared with the dose calculated based on the individual patient’s ICR and the simultaneous suspension of basal insulin for 2 h. Normal bolus was defined as the prandial insulin dose calculated based on the individual’s ICR. 

The primary outcome of the study was the capillary blood glucose level 90 min after administration of the prandial bolus, based on the glucometer data. Secondary endpoints were: (1) capillary blood glucose level 30, 60, 90, 120, 150, and 180 min after administration of the prandial bolus (glucometer data); (2) number of hypo- and hyperglycemic episodes (glucometer and CGM data, separately); (3) glycemic rise (GR) from the baseline to the maximum glucose level (CGM data); (4) peak glucose (PG) level within 180 min after the prandial bolus administration (CGM data); (5) time to peak glucose level (CGM data); (6) mean amplitude of glycemic excursion, MAGE (CGM data); (7) time in the postprandial glucose range between 70–180 mg/dL (3.9–10.0 mmol/L), TIR (CGM data); (8) area under the blood glucose curve, AUC (CGM data). Two types of hypoglycemic episodes were distinguished: with the lowest result (1) TBR1, between 54 and 69 mg/dL (3.0–3.8 mmol/L) or (2) TBR2, below 54 mg/dL (<3.0 mmol/L). Similarly, hyperglycemia was pointed out as with highest result (1) TAR1, between 181 and 250 mg/dL (10.0—13.9 mmol/L) or (2) TAR2, over 250 mg/dL (>13.9 mmol/L). Each type of episode was analysed separately. If the data from CGM indicated the glucose level below or above threshold in consecutives measurements, we considered it as one episode of hypoglycemia or hyperglycemia until the value in range was reached. Every measurement performed by the glucometer was analyzed as a separate episode. We did not include hyperglycemic episodes as an endpoint in the study protocol. After completing the data, we decided that this is an important parameter that we should take into consideration and conducted an analysis of the hyperglycemic episodes.

### Statistical Analysis

The sample size was estimated based on calculations performed using StatsDirect statistical software (V.3.1.4, StatsDirect, Chesire, UK). 72 participants were required to demonstrate a difference of 30 mg/dL (1.7 mmol/L) and a standard deviation (SD) of 41, SD for observations within treatment being 58 at the 90th minute of the study (the primary endpoint), assuming α = 0.05, power of 80% and a 20% dropout rate.

The study group was described with appropriate descriptive statistics. The number of missing data was presented for each variable. The normality of distribution was verified based on Shapiro-Wilk test. The characteristics of the patients and the outcomes were presented using mean and standard deviation for normally-distributed variables, median and quartiles for continuous non-normally-distributed variables, numbers, and percentages for discrete variables. The insulin sensitivity factor (ISF) was calculated using the 1800 rule.

The results obtained after the administration of the super bolus and normal bolus were compared using the paired Student *t*-test in case of normal distribution and the Wilcoxon matched-pairs signed-rank test if outcomes were non-normally distributed. The outcomes were presented as differences in means or medians for the continuous data and with odds ratios for nominal variables, both with a 95% confidence interval. All tests were two-tailed with significance level of α = 0.05. 

Intention-to-treat analysis was performed as a primary approach, with additional per-protocol analysis as a sensitivity analysis. In the *per-protocol* analysis, eight patients were excluded, seven due to CGM system failure (sensor detachment, interruption of data transmission, missing readings, very large discrepancies in results from the glucometer and CGM system) and one patient failure to measure glucose level at 180th minute with a glucometer. Additionally, linear regression analysis with the baseline value of the outcome as a covariate was used to compare the treatment groups. The clinical inference was based on 95% confidence intervals for the regression coefficient for the treatment effect. If the 95% confidence interval did not include 0, we concluded that the difference between the treatment groups was clinically significant. The area under the blood glucose curve for each patient was estimated using cubic splines.

Statistical analysis was carried out in StataCorp. 2017. *Stata Statistical Software: Release 15*. StataCorp LLC, College Station, TX, USA. Figures were prepared using GraphPad Prism 6.00 (GraphPad Software, La Jolla, CA, USA).

## 3. Results

**Figure 1:**
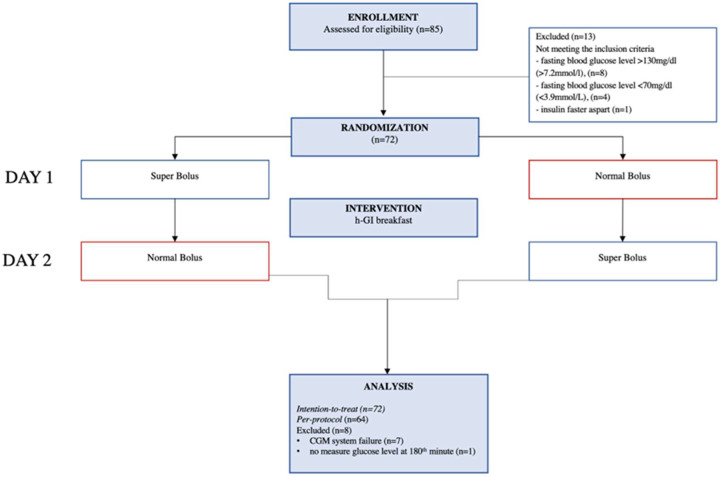
Study flow diagram.

The study flow diagram presents Figure 1. In this study, we included 72 teenagers with a T1D duration of about 6 years, with an equal gender distribution. There was no predominance concerning the type of infusion set and the localization of cannulas. Most participants were insulin aspart users (NovoRapid^®^, NovoNordisk, Bagsværd, Denmark). The baseline characteristics of the study group are presented in Table 1. See Figure S1 in the Supplementary Material for the linear regression graph of the insulin-to-carbohydrate ratio (ICR) as a function of the age. The graph shows the correlation of the increase in the ICR with the increase in participants’ age.

**Figure 2:**
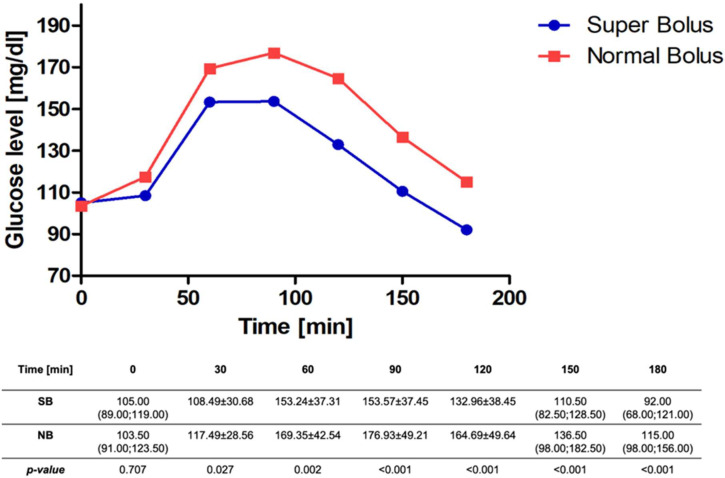
Glucose levels after super bolus and normal bolus—values measured by glucometer.

**Figure 3:**
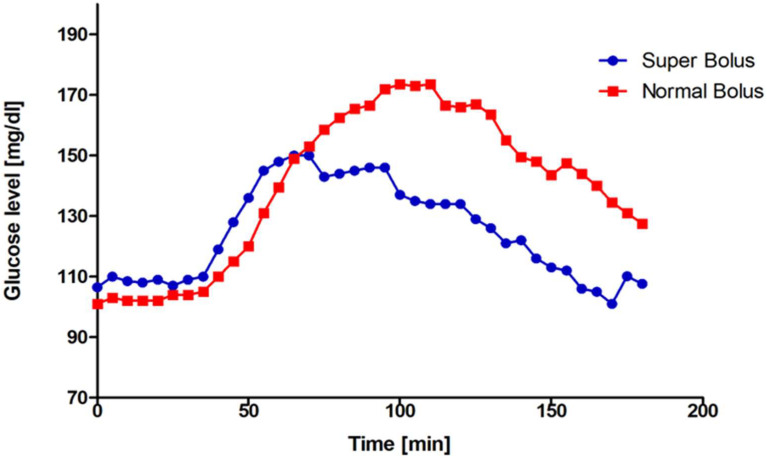
Glucose levels after super bolus and normal bolus—values from CGM.

**Figure 4:**
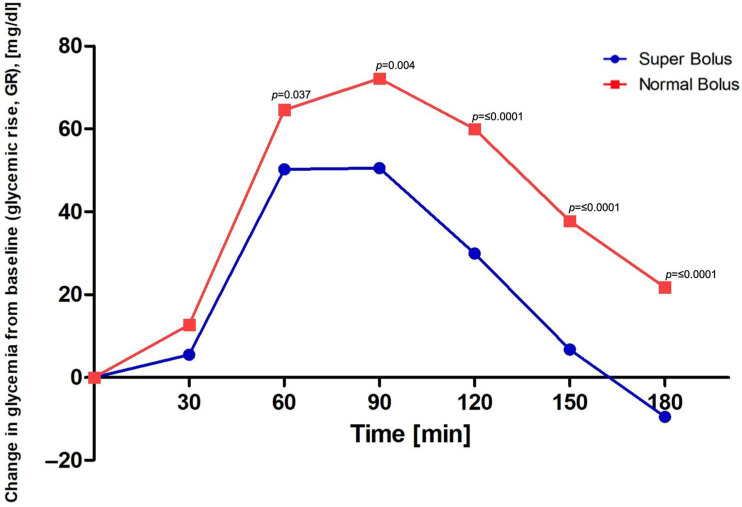
Change in glycemia (glycemic rise, GR) from baseline value.

We found a significant difference in the primary outcome-capillary blood glucose level 90 min after the administration of the prandial bolus. Patients who received SB had lower glucose levels 154 ± 37.4 mg/dL (CI_95_[144.77; 162.37]) vs. 177 ± 49.2 mg/dL (CI_95_[165.37; 188.49), MD = −23, *p* < 0.001. In general, significantly lower values were confirmed for each time point excluding the baseline glucose values in the super bolus group. Figure 2 presents measured by glucometer values after SB and NB implementation. Figure 3 shows analogue data obtained from CGM. The median time in range 70–180 mg/dL (3.9–10.0 mmol/L) was higher in the SB group: 165 min (135.0; 180.0), 91.67% vs. 152.5 min (103.8; 180.0), 84.72%, *p* = 0.001. The glucose values were also more stable in this group: the median rise of glycemia (glucose change from baseline) was significantly lower after SB administration (58 mg/dL (40.0; 93.0) vs. 90 mg/dL (40.0; 93.0), *p* ≤ 0.001). The median GR at each time point is shown in Figure 4. The PG was significantly higher for the NB group 188 mg/dL (146.0; 219.0) than for SB 158 mg/dL (134.0; 193.0), *p* ≤ 0.001. The time to reach PG was similar in both groups (SB 95 min (70.0; 120.0), NB 105 min (85.0; 125.0)), *p* = 0.067. Greater glycemic variability occurred in SB, MAGE 30.44 vs. 22.89, *p* = 0.001. SB application caused over 11% reduction in AUC, 22,626.54 ± 5877.49 for SB and 25,437.49 ± 6880.06 for NB, *p* ≤ 0.001. Most participants did not experience hypoglycemic episodes during the study period (CGM data: 71.01% SB, 81.43% NB *p* = 0.030). No episodes of serious hypoglycemia as neurogenic symptoms or cognitive dysfunction were noted. More hypoglycemic episodes were noted in the SB group and the time spent in hypoglycemia (time below range) was higher per participant: SB 5.94 min vs. 2.79 min, *p* = 0.012). The frequency of hypoglycemic episodes was 0.36/per participant in the SB group and 0.21/per participant in the NB group, *p* = 0.029 (data from CGM). Almost 90% of the episodes were threshold value for initiating treatment for hypoglycemia (69–54 mg/dL). If hypoglycemia appeared, it was in the final phase of the study, at the end of the observation period (SB 145 min (35.0; 160.0), NB 155 min (30.0; 165.0), *p* = 0.889) in both groups. We did not note differences concerning the time in hypoglycemia below 54 mg/dL, *p* = 0.098.

**Figure 5:**
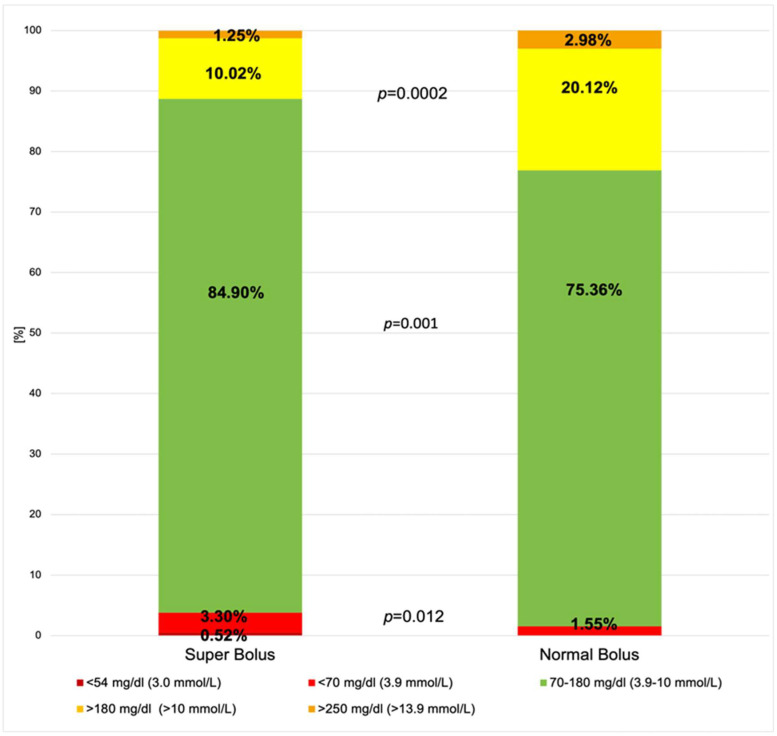
Comparison between study groups: mean time in range, above range, and below range.

More hyperglycemic episodes were noted in the NB group; 59.72% participants in this group had an episode of hyperglycemia. Participants after NB also spent over twice as much time in hyperglycemia (*p* = 0.0002). We did not find any significant differences between the groups concerning time spent in hyperglycemia in the range above 250 mg/dL, *p* = 0.293. Figure 5 presents comparisons between the mean time in ranges in both groups. A detailed analysis of hypo- and hyperglycemic episodes is shown in Table 2. 

There was a significant positive linear relationship between both groups confirmed for capillary blood glucose levels, GR, PG, time to PG, AUC, MAGE, TIR excluding glucose baseline level (*p* = 0.451). In the per protocol approach, a regression model was not significant for glucose baseline level (*p* = 0.289) as well as PG (*p* = 0.289)

See Table S1 in the Supplementary Material for per protocol approach of study outcomes.

## 4. Discussion

To the best of our knowledge, this is the first clinical study to evaluate whether SB is a better strategy than NB for preventing postprandial hyperglycemia after a h-GI meal. The key finding of this study is the fact that proposed SB is an effective and safe strategy. We found lower glycemic values not only in the primary endpoint (90th minute), but also during the entire observation period in the SB group.

Our findings are consistent with other earlier reports concerning SB that it is a potential solution to h-GI meals. The problem is that there is no single definition. The SB concept was conducted by Walsh and Roberts as a novel manner of insulin delivery for future smart insulin pumps. They suggested suspending basal insulin for 2–4 h and adding this amount of insulin to the pre-meal bolus [22,23]. Some authors suggest the same solution that the total amount of insulin is unchanged, and the basal insulin is removed over the next 2 h [15,16]. Bondia et al. found that the unchanged load of insulin is an effective solution for meals containing up to 50 g of carbohydrates [18]. Based on an in silico model, the amount of meal bolus should be increased by 50–60% with the subsequent basal decrease depending on the carbohydrate content but lasting for a maximum of 3 h [18]. In our case, for a 60 g meal, it should be an 80% basal reduction for 3 h [18]. Analyzing the obtained results, it seems that the mentioned combination would be the most effective. In this study group, some patients after SB bolus experienced mild hypoglycemia, about 2.5–3 h after bolus administration. It suggests the need for longer than 2 h basal insulin reduction.

We can compare our study results to those of Groele et al., which used a 30% increased ICR for breakfast cereals, however included a smaller group of patients and used different methodology [14]. Our findings are consistent that h-GI meals require an additional dose of insulin to reduce postprandial hyperglycemia. SB administration caused the higher reduction of glycemic rise and provided the stable and in range glycemic values in comparison to the 30% increased meal bolus [14]. We also noted a reduction in AUC, which is the evidence of whole glucose excursion which was not noted if the ICR was increased by 30% [14]. The study conducted by Rosales et al. evaluates the in-silico model of the automatic SB based on insulin on board level [17]. They found that the higher the carbohydrate content, the more difficult it is to control glycemia. After a single meal containing 50 g of carbohydrates, automatic SB led to TIR 93.53% vs. 89.67% for standard treatment; 75 g TIR automatic SB 89.51% vs. 81.9% for standard treatment [17]. Our results are similar to those from the in-silico study, but we noted a slightly higher difference between standard treatment and SB. Participants who have applied SB achieved 91.67% and 84.72% for NB. 

When it comes to hypoglycemic episodes, we noted that the SB caused a greater rate than the NB. We obtained more hypoglycemic episodes when the glucometer data was analyzed than CGM. This was due to a different technique for counting events. In the glucometer-derived data, each measurement below 70 mg/dL (3.9 mmol/L) was treated as a separate hypoglycemic episode, while in the CGM data as a single episode were treated glycemic values below range in subsequent measurements until they reached the value of ≥70 mg/dL (3.9 mmol/L). The frequency of hypoglycemic episodes was similar to that obtained when the meal bolus was increased by 30% to a meal with a high glycemic index [14]. The important fact is that most participants did not experience hypoglycemia and most episodes were at the alert hypoglycemic level. We observed that hypoglycemia occurred near the end of study period (2.5–3 h after prandial bolus). It indicates the excess of insulin on board and seems that proposed suspension basal insulin for 2 h was too short. It would be reasonable to remove the base insulin for a longer time, for example 3 h. It is worth emphasizing that after SB, the insulin on board (IOB) level is high, which increases the risk of hypoglycemia. Thus, physical activity should be undertaken with caution, taking into account the need to consume an additional amount of carbohydrates or postponed for at least 2 h after bolus administration [24].

Considering the use of different insulin formulations, there is a potential for faster aspart to reduce postprandial glycemia. Data from the pediatric population indicated that after a standardized meal, glycemic excursions were reduced among children (6–11 years), without effect among adolescents [11]. In the context of h-GI meals, we can refer to a study conducted by Cutruzzolà et al. [13]. The authors compared insulin faster aspart and aspart given with a high-glycemic-index meal among adult participants and showed no differences in postprandial glycemic values and glycemic rise. They found differences concerning the AUC in favor of insulin faster aspart [13].

Vetrani et al. found that among people using automated hybrid closed-loop (AHCL) systems after breakfast, there was observed an early rise in blood glucose, peaking after just 1 h [25]. They also found that daily TIR was significantly associated with breakfast TIR, which leads us to pay special attention to proper glycemic control, especially in the morning. It is not clear if the AHCL system can compensate the SB effect, but a 50% increased dose of prandial insulin seems to be a very good solution for this group of patients for h-GI breakfast. Theoretically, the system should suspend the insulin delivery as long as needed to minimize the risk of hypoglycemia, which occurred more frequently after SB administration in our study group (on sensor-augmented pump therapy). 

The strength of our study is its well-planned methodology (randomization, double blinding) and well-selected, large, and homogenous group of patients. This is evidenced by the publication of the study protocol before the study began [20]. We performed a crossover trial, so every patient performed both the intervention (SB) and the control (NB) under the same conditions (the same meal was consumed at the same hour with fasting normoglycemia). It was proceeded by a run-in period so insulin doses and ICRs were properly matched. We have correctly established the capillary blood glucose level 90 min after prandial bolus as a primary end point according to previous reports in which it has been shown that the glycemic value after h-GI products is the highest at this point among people with T1D [1,5,6,7]. Our results are consistent with these observations; PG was about the 100th minute of observation time regardless of the applied meal bolus. The weakness of this study is its short observation period after the prandial bolus (3 h). This time should be longer to observe the consequences of basal removing. We performed only the single meal scenario, and all participants ate the same portion, independently of their weight. We could also add the arm with different parameter of SB, f. ex. with longer basal insulin suspension. 

The purpose of our study was to encourage diabetic patients not to consume high glycemic index products. We would like to highlight that high glycemic index and glycemic load products have been shown to be primarily responsible for postprandial hyperglycemia and reduced TIR [25,26]. Our study is an attempt to answer a common clinical problem, especially among the youngest groups of patients, where it is difficult to properly balance meals.

There is no doubt that this data supports the use of the super bolus for h-GI products, but further studies are needed to precisely determine how much and for how long the base insulin should be suspend or reduce. This study can also be repeated using insulin with different pharmacokinetics, like faster insulin aspart. 

## 5. Conclusions

Super bolus is an effective and safe strategy to avoid postprandial hyperglycemia.

This kind of bolus leads to a higher time in range 70–180 mg/dL (3.9–10.0 mmol/L) after the high GI meal consumption.

Hypoglycemic episodes occurred more often after the super bolus in comparison to the normal bolus. Most hypoglycemic episodes were at the alert level and occurred near the end of the observation period. Therefore, it seems reasonable to extend the time of the basal insulin suspension, f.ex. to 3 h. Further studies are needed to find the best combination of the increased dose of meal bolus and basal insulin reduction.

## Figures and Tables

**Table 1:** Baseline characteristics of study group.

Characteristic	N	n (%)/Mean ± SD/Median (Q1;Q3)
Sex, n (%)	72	
Female		37 (51.39)
Male		35 (48.61)
Age (years)^1^	72	14.97 (12.96;16.30)
Duration of disease (years)^1^	72	5.91 (2.79;9.44)
BMI (kg/m^2^)^1^	72	20.77 (18.63;22.91)
BMI Z-score^1^	72	−0.07 (−0.71;0.57)
TDD/kg (u/kg)^2^	72	0.82 ± 0.25
Base/kg (u/kg)^2^	72	0.34 ± 0.12
HbA1c (%)^2^	72	8.35 (7.45;9.30)
Breakfast’s ICR (u)^2^	72	1.61 ± 0.56
ISF (mg/dL)^1^	72	37.66 (29.75;50.28)
Insulin type, n (%)	72	
Novo Rapid^®^, Novo Nordisk		29 (40.28)
Humalog^®^, Eli Lilly		13 (18.06)
Apidra^®^, Sanofi		10 (13.89)
Liprolog^®^, Eli Lilly		19 (26.39)
Lispro^®^, Sanofi		1 (1.39)
Infusion set type, n (%)	72	
Teflon cannula		39 (54.93)
Metal cannula		32 (45.07)
Infusion set localization, n (%)	72	
Abdomen		19 (26.76)
Arm		18 (25.35)
Thigh		23 (32.39)
Buttock		11 (15.49)

Data presented as median (Q1;Q3)^1^ or mean ± SD^2^ unless otherwise indicated. Base/kg—basal insulin dose calculated per kg of body mass, BMI—Body Mass Index, TDD/kg—daily insulin dose calculated per kg of body mass, ICR—insulin-carbohydrate ratio (defined as a dose of insulin necessary to cover 10 g of carbohydrates), ISF—insulin sensitivity factor (calculated using 1800 rule), u—unit.

**Table 2:** Total number of hypo- and hyperglycemia episodes in the study groups.

Total Number of Episodes	Super Bolus Group	Normal Bolus Group	*p*-Value
TBR2 (G)	9 (0.13/pp)	3 (0.04/pp)	**0.041**
TBR2 (CGM)	4 (0.06/pp)	0	0.072
TBR1 (G)	33 (0.45/pp)	14 (0.20/pp)	**0.003**
TBR1 (CGM)	25 (0.36/pp)	15 (0.21/pp)	**0.029**
TAR1 (G)	45 (0.63/pp)	99 (1.38/pp)	**<0.0001**
TAR1 (CGM)	22 (0.32/pp)	38 (0.54/pp)	**0.001**
TAR2 (G)	1 (0.01/pp)	15 (0.21/pp)	**0.022**
TAR2 (CGM)	3 (0.04/pp)	6 (0.09/pp)	0.299

For CGM data: Super Bolus Group n = 69, Normal Bolus Group n = 70; For glucometer data n = 72; CGM—continuous glucose monitoring system data, G—glucometer data, pp—per participant; TBR1: 69–54 mg/dL; TBR2 < 54 mg/dL; TAR1: 181–250 mg/dL; TAR2 > 250 mg/dL.

## Data Availability

Data available on request due to ethical reasons.

## Supplementary Materials

The following supporting information can be downloaded at: https://www.mdpi.com/article/10.3390/nu16020263/s1, Figure S1. Linear regression model of the insulin-to-carbohydrate ratio (ICR) as a function of the age. Table S1. Study outcomes comparisons between treatment groups- per protocol approach. Data presented as median (Q1;Q3)^1^ or mean ± SD^2^ unless otherwise indicated.
