# Supplementary material for: Super Bolus—A Remedy for a High Glycemic Index Meal in Children with Type 1 Diabetes on Insulin Pump Therapy?—A Randomized, Double-Blind, Controlled Trial
Source: Nutrients. 2024 Jan 16;16(2):263. doi: 10.3390/nu16020263 (PMC10818731; doi:10.3390/nu16020263)
Supplement: Supplementary file 1 [file nutrients-16-00263-s001.zip › SB_Suppl Figure S1.pdf]

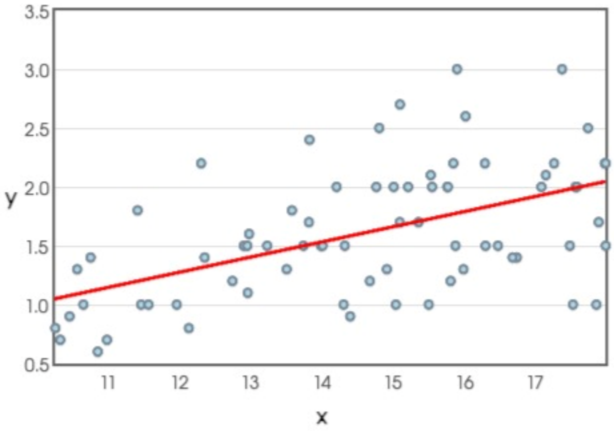

Figure S1. Linear regression model of the insulin-to-carbohydrate ratio (ICR) as a function of the age. X- age [years], Y- ICR [u]
