# Supplementary material for: Super Bolus—A Remedy for a High Glycemic Index Meal in Children with Type 1 Diabetes on Insulin Pump Therapy?—A Randomized, Double-Blind, Controlled Trial
Source: Nutrients. 2024 Jan 16;16(2):263. doi: 10.3390/nu16020263 (PMC10818731; doi:10.3390/nu16020263)
Supplement: Supplementary file 1 [file nutrients-16-00263-s001.zip › SB_Suppl Table S1.pdf]

PER PROTOCOL analysis

Characteristic	Super Bolus (n=64)	Normal Bolus (n=64)	p-value
CBGL 0 min (mg/dl)[1]	105.00 (92.00;120.50)	104.00 (90.00;122.50)	0.965
CBGL 30 min (mg/dl)[2]	109.61±31.40 95%CI[101.77 to 117.45]	118±28.93 95%CI[110.77 to 125.23]	0.049
CBGL 60 min (mg/dl)[2]	154.58±38.06 95%CI[145.07 to 164.08]	171.41±43.68 95%CI[160.50 to 182.32]	0.002
CBGL 90 min (mg/dl)[2]	154.83±38.78 95%CI[145.14 to 164.51]	179.88±50.70 95%CI[167.21 to 192.54]	<0.001
CBGL 120 min (mg/dl)[2]	133.69±39.40 95%CI[123.84 to 143.53]	168.2±50.70 95%CI[155.54 to 180.87]	<0.001
CBGL 150 min (mg/dl)[1]	111.08±37.05 95%CI[101.82 to 120.33]	145.64±50.55 95%CI[133.01 to 158.27]	<0.001
CBGL 180 min (mg/dl)[1]	91.50 (68.00;121.00)	115.00 (98.00;156.00)	<0.001
PG (mg/dl)1	159.00 (135.50;191.50)	195.00 (146.50;222.50)	<0.001
GR (mg/dl) [1]	58.00 (40.00;87.50)	92.00 (53.00;107.00)	<0.001
Time to PG (min)[1]	99.53±41.64	107.81±35.84	0.166
MAGE (mg/dl)	23159.57±5332.45	26103.38±6483.57	<0.001
Time in range between 70-180 mg/dl (min)[1]	165.00 (137.50;180.00)	130.00 (100.00;180.00)	<0.001
Total AUC[2]	23159.57±5332.45	26103.38±6483.57	<0.001

Table S1. The main study outcomes- per protocol approach. Data presented as median (Q1;Q3)[1] or mean±SD[2] unless otherwise indicated.

AUC- area under the blood glucose curve, CBGL- Capillary Blood Glucose Level, CI – confidence interval, GR- glycemic rise, MAGE- mean amplitude of glycemic excursions, PG- peak glucose.

**Number of hypoglycemia episodes**	Number of participants (%), based on **CGM** data		Number of participants (%), based on **glucometer** data	
	Super Bolus group	Normal Bolus group	Super Bolus group	Normal Bolus group
0	46 (71.88)	53 (82.81)	33 (51.56)	54 (84.38)
1	14 (21.88)	9 (14.06)	26 (40.63)	9 (14.06)
2	3 (4.69)	2 (3.13)	5 (7.81)	1 (1.56)
3	1 (1.56)	0 (0.0)	0 (0.0)	0 (0.0)
	p=0.054		p<0.001	
